# Supplementary material for: PHRF1 promotes the class switch recombination of IgA in CH12F3-2A cells
Source: PLoS One. 2023 Aug 4;18(8):e0285159. doi: 10.1371/journal.pone.0285159 (PMC10403053; doi:10.1371/journal.pone.0285159)
Supplement: S1 File — (DOCX) [file pone.0285159.s001.docx]

**Supporting Information**

**PHRF1 promotes the class switch recombination of IgA in CH12F3-2A cells**

Jin-Yu Lee^1,*^, Nai-Lin Chou^1,*^, Ya-Ru Yu^2^, Hsin-An Shih^1^, Hung-Wei Lin^1^, Chine-Kuo Lee^2^, and Mau-Sun Chang^1,3,**^

^1^Institute of Biochemical Sciences, National Taiwan University, Taipei, Taiwan

^2^Graduate Institute of Immunology, National Taiwan University College of Medicine, Taipei, Taiwan

^3^Institute of Biological Chemistry, Academia Sinica, Taipei, Taiwan


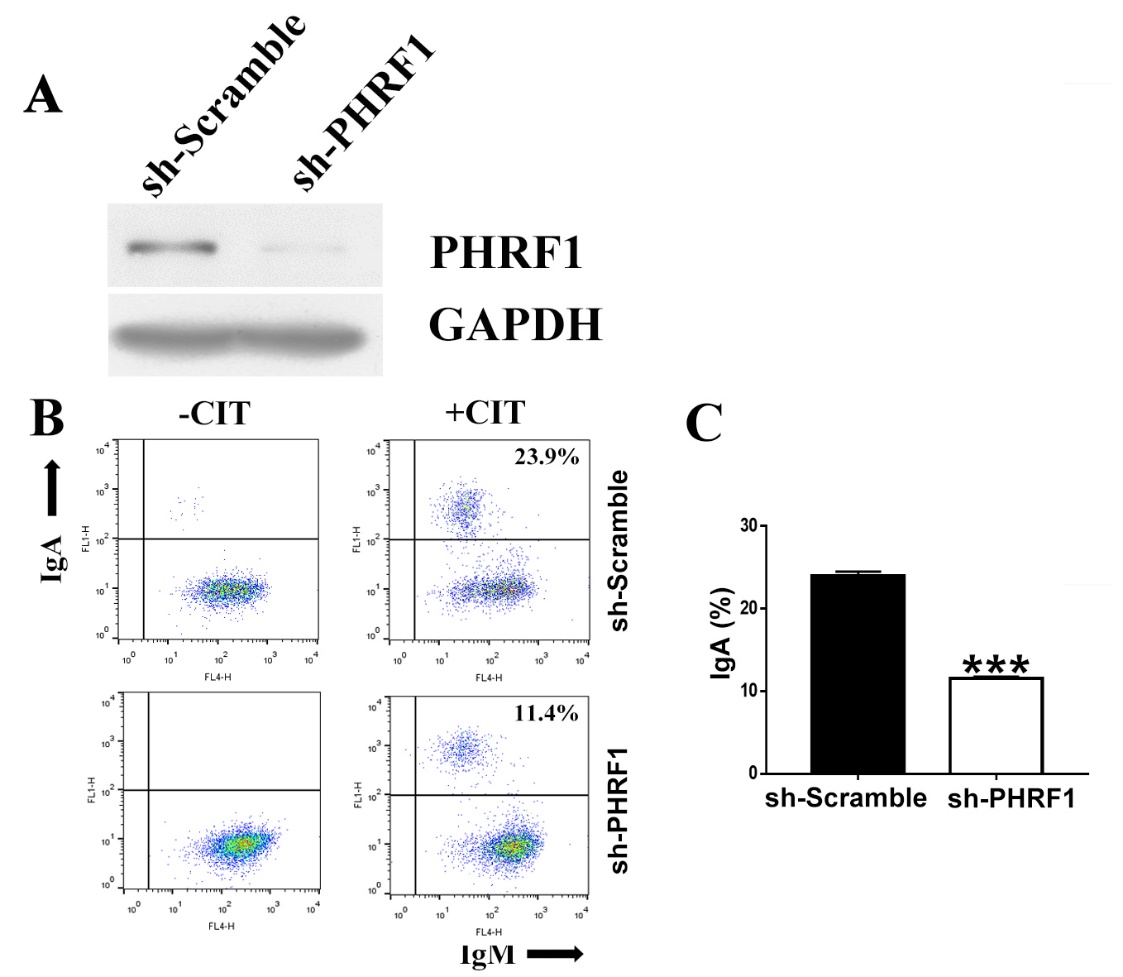


**S1 Fig. Knockdown of PHRF1 by shRNA decreased IgA production in CH12F3-2A cells.** (A) Scramble and PHRF1 shRNAs were transduced into CH12F3-2A cells and immunoblotting analysis was conducted by indicated antibodies. All Western blots were processed in identical conditions and cropped from S9 Fig. (B) Representative images of the IgA population were identified by flow cytometry. (C) The percentage of IgA switching was quantified in the scramble and PHRF1 silencing CH12F3-2A cells treated with CIT. Three independent experiments were presented as mean ± SD. **, P < 0.01 by Student’s *t*-test.

**
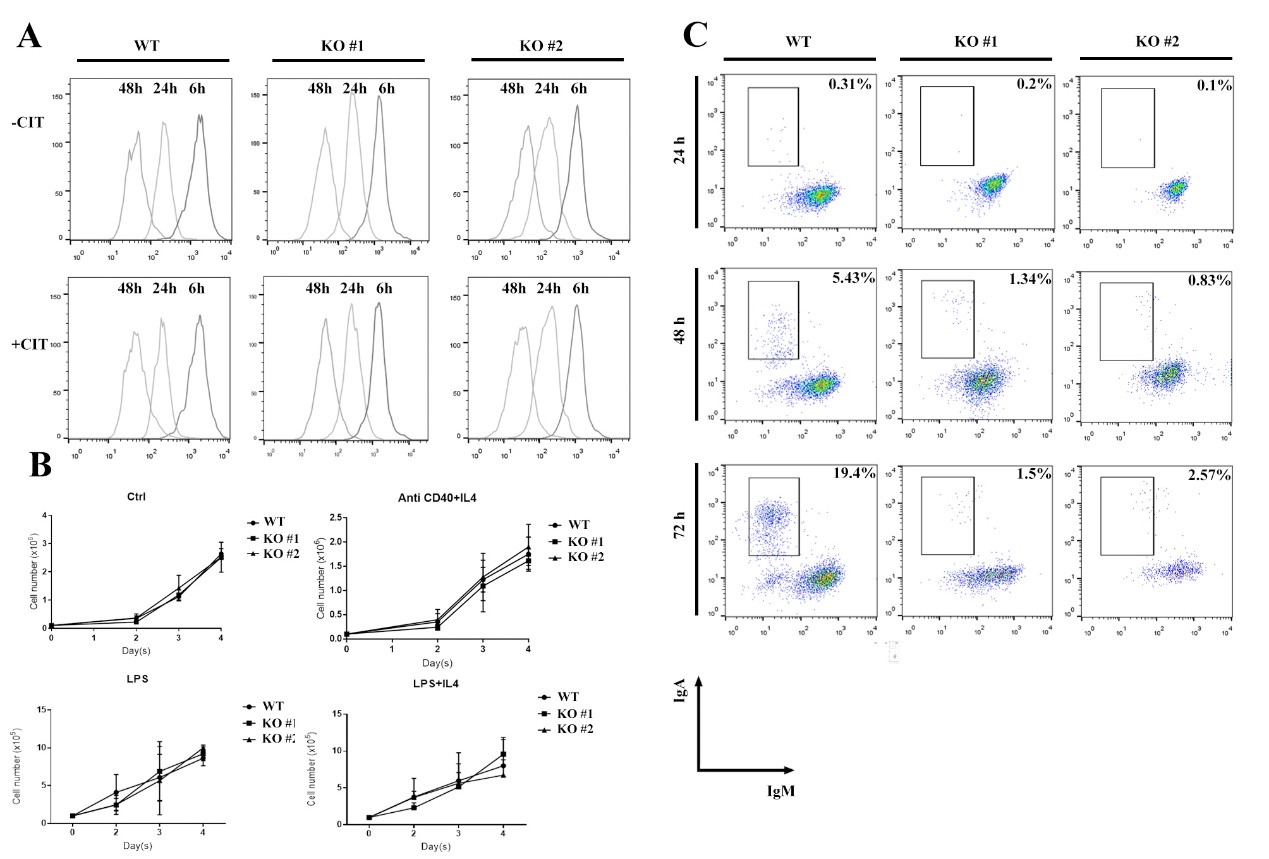
**

**S2 Fig.** **The proliferation of control and PHRF1 KO CH12F3-2A cells.** (A) Analysis of cell proliferation of WT, PHRF1 KO#1, and KO#2 cells was measured using CFSE labeling. Histograms showed CFSE dye dilution at 6, 24, and 48 h post-CFSE labeling. (B) 2 x 10^5^ WT, PHRF1 KO#1, and KO#2 cells were incubated under different conditions. Proliferation curves of three independent experiments were measured. Each point represented the mean ± SD from three independent experiments. Note that there is no significant difference in control and PHRF1 KO cells. (C) The time course of WT, KO#1, and KO#2 cells exposed to CIT stimulation was carried out. Representative images of IgA switching with CIT were shown by flow cytometry.


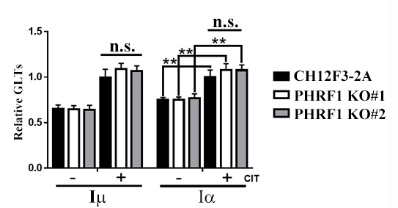


**S3 Fig. Germline transcription (GLT) in PHRF1 deficient cells.** Iμ and Iα transcripts in WT, PHRF1 KO#1, and KO#2 CH12F3-2A cells were measured by the quantitative PCR and normalized to the level of HPRT. Primers for Iμ were 5’-AAGGCTTCCAAAGTCACGTTCC-3’ and 5’-GGAAGACATTTGGGAAGGACTG-3’. Primers for Iα were 5’-GTGAAAGACTACCTGCAGGTCA-3’ and 5’-GTGGGAGTGTCAGTGGGTAGAT-3’. Three independent experiments were presented as mean ± SD. Note that GLTs were increased post-CIT treatment. ** P < 0.01 by Student’s *t*-test. n.s., not significant.


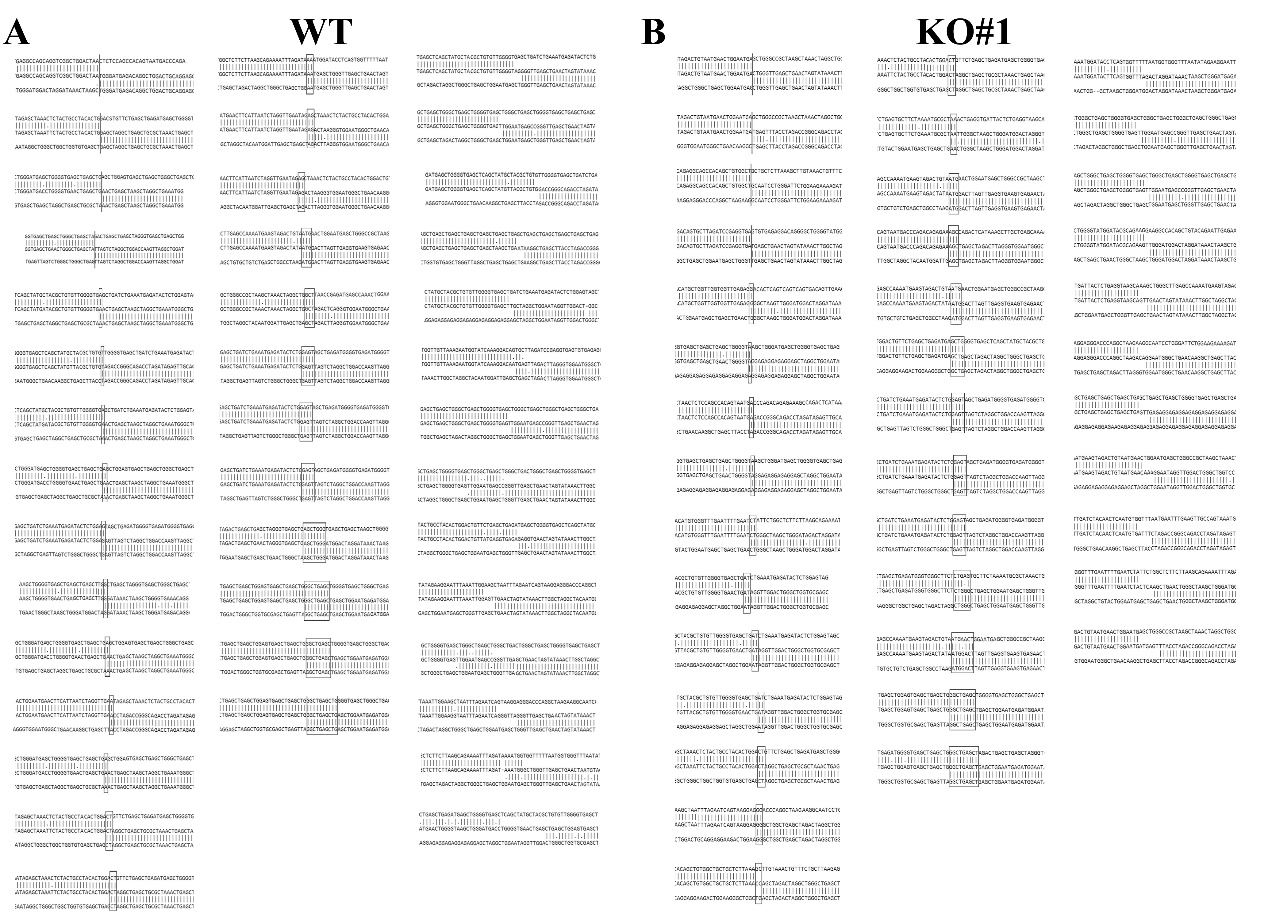


**S4 Fig. Alignment of microhomology in Sμ-Sα joining.** Junctional sequences from WT (n = 41) and PHRF1 depleted CH12F3-2A cells (n = 37) were examined. Sμ and Sα joining sequences were listed on the top and bottom, respectively. Microhomology was marked in box. Vertical lines indicate direct joins. **
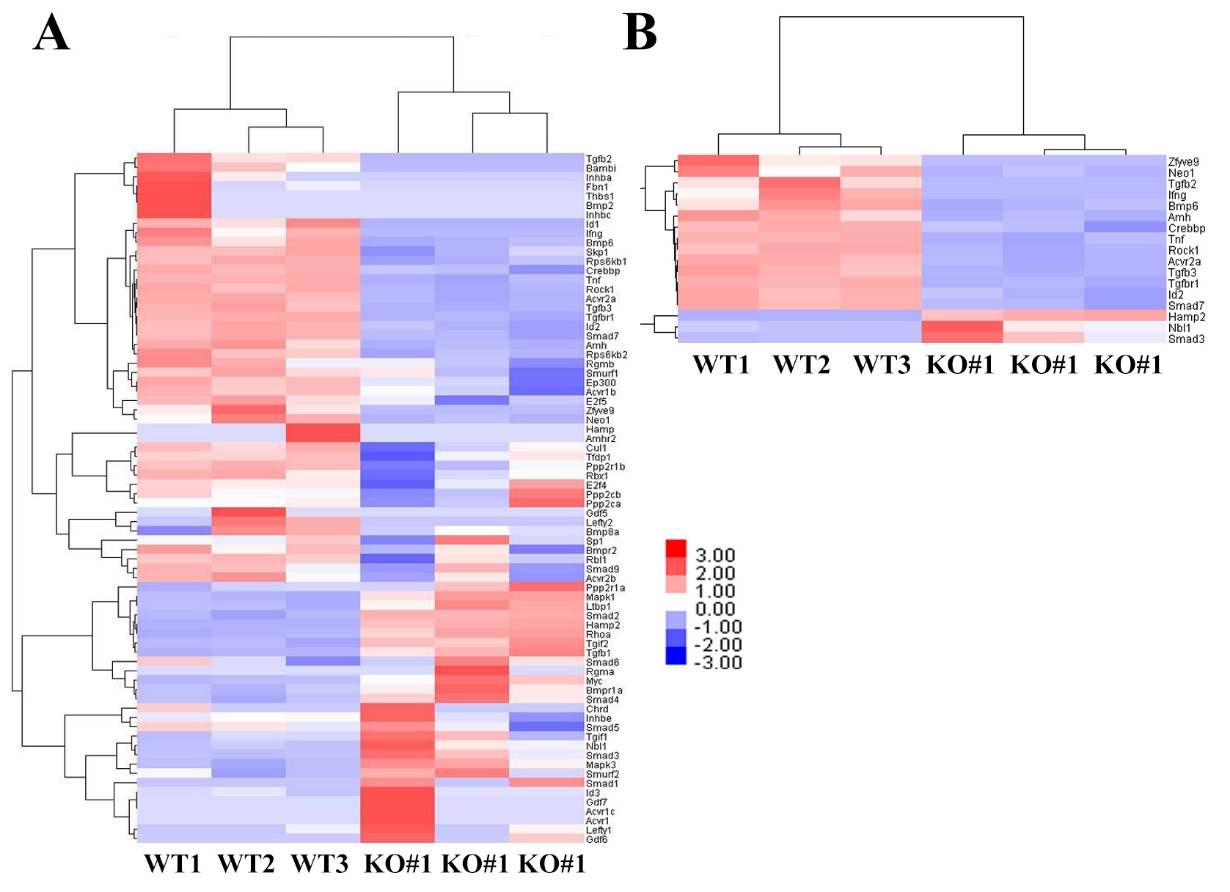
**

**S5 Fig. Heatmap clustering of TGF-β signaling in WT and PHRF1 KO#1 cells.** (A) The heatmap was generated by clustering the whole components of TGF-β signaling (KEGG#04350, n = 3 per group). Down-regulated genes are shown in blue shades and up-regulated genes are shown in red shades. (B) The heatmap by clustering the fold change (FC) >2 and FC <0.5 in TGF-β signaling (n = 3 per group).

**
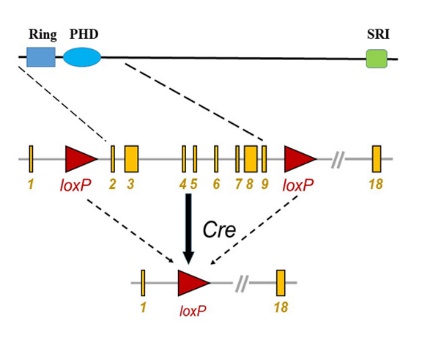
**

**S6 Fig. Conditional knockout of *PHRF1* gene in CD19-Cre mice.** Schematic representation of the mouse *PHRF1* gene flanked by two loxP sites to delete the Ring and PHD domains in the presence of Cre recombinase.


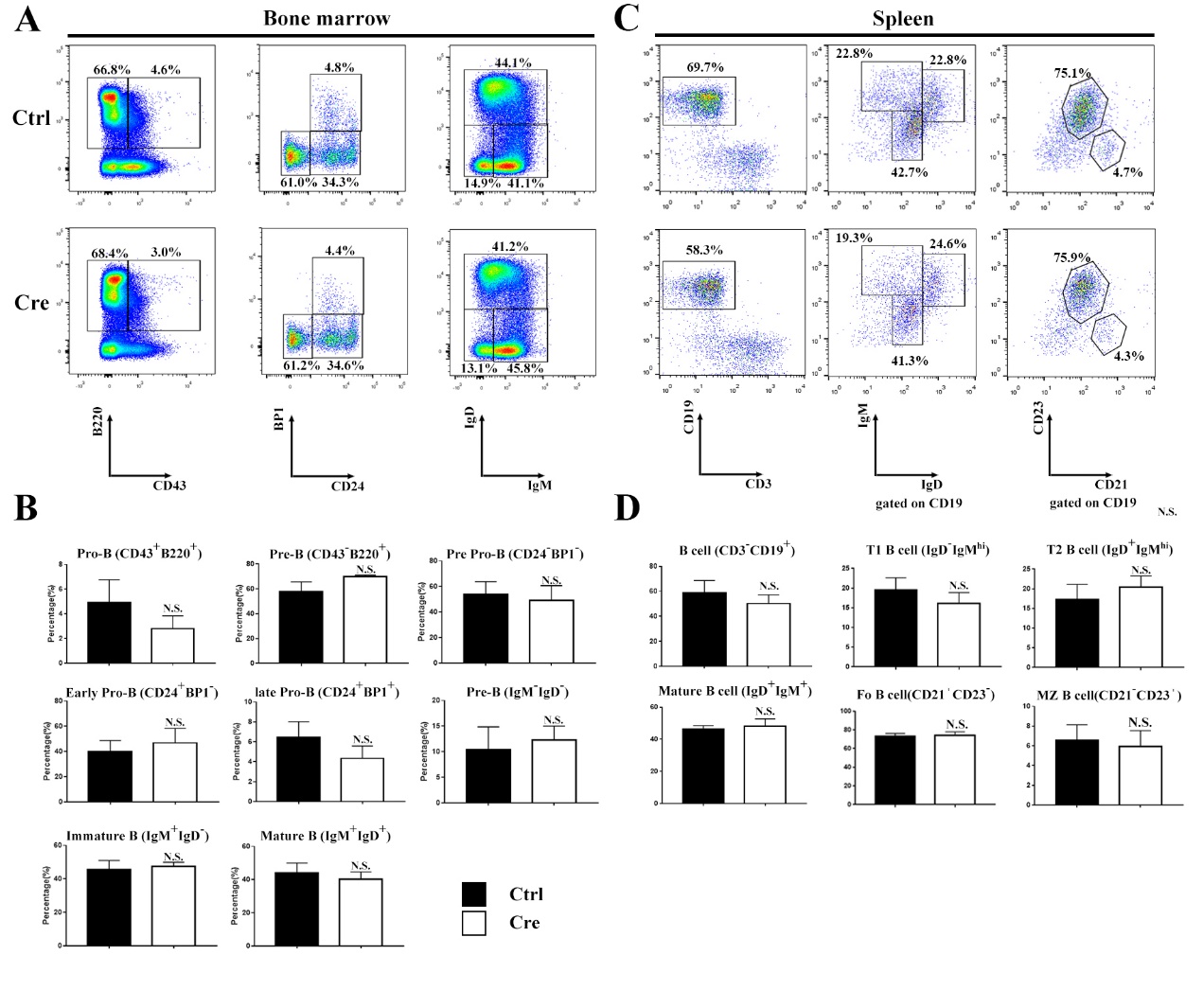


**S7 Fig. PHRF1 ablation did not affect B cell development.** (A, C) Bone marrow and spleen cells were isolated from 8-week-old control and Cd19^Cre/+^ PHRF1^f/f^ mice. Cells were immunostained with indicated antibodies for different stages in B cell development. (B, D) Quantitative results for (A, C) were represented from three independent experiments ± SD. n.s., not significant by Student’s *t*-test.


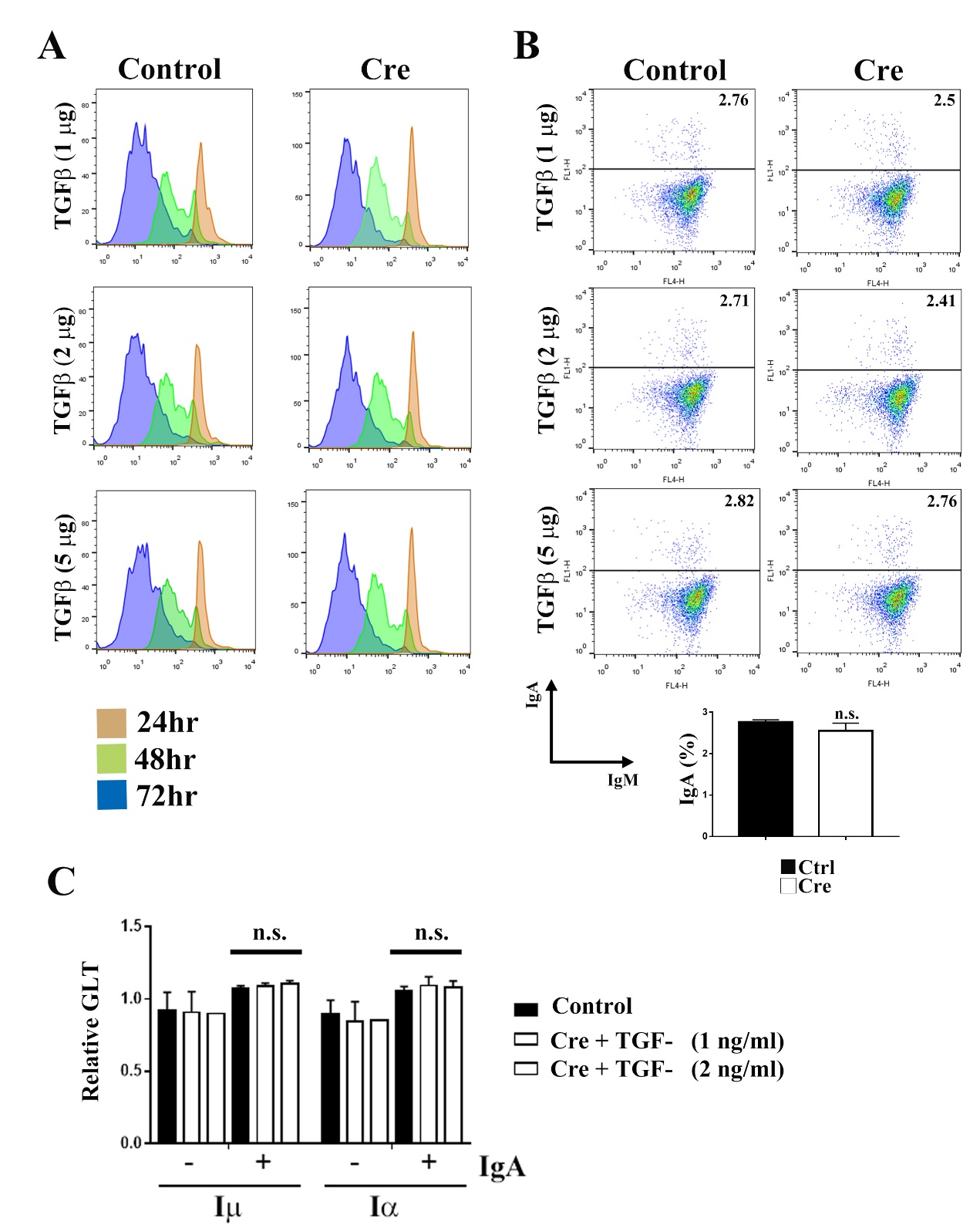


**S8 Fig.** **The proliferation, IgA production, and GLT in response to TGF-β in primary splenic B cells.** (A) Cell proliferation in different doses of TGF-β was measured using CFSE labeling in control and PHRF1 KO B cells. Histograms showed CFSE dye dilution at 24, 48, and 72 h post-CFSE labeling. (B) IgA switching in different doses of TGF-β was determined by flow cytometry. Quantitative results were measured by three independent experiments. n.s., not significant by Student’s *t*-test. (C) Iμ and Iα transcripts in control and Cd19^Cre/+^ PHRF1^f/f^ B cells were measured by the RT-qPCR and normalized to the level of HPRT. n.s., not significant by Student’s *t*-test.


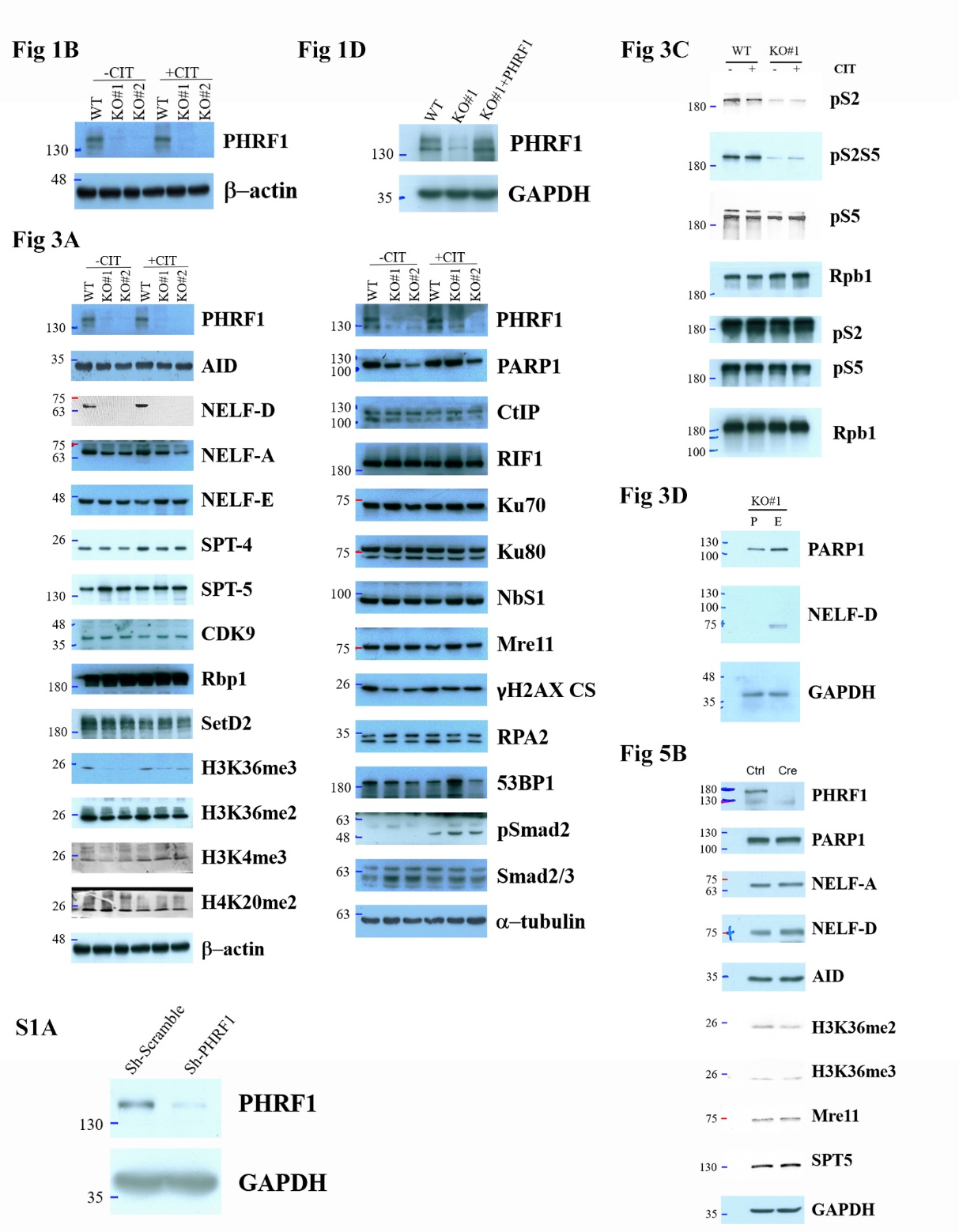

**S9 Fig. Uncropped images for all gels and Western blots.**

**S1 Table. List of antibodies used in this study.**

| NELF-A | Santa Cruz | SC-365004 |
| --- | --- | --- |
| NELF-D | Santa Cruz | SC-393972 |
| NELF-E | Santa Cruz | SC-377052 |
| Spt4 | Santa Cruz | SC-515238 |
| Spt5 | Santa Cruz | SC-133217 |
| PARP1 | Santa Cruz | SC-7150p |
| phospho-Smad2 (S465/S467) | Cell Signaling | #3108 |
| phospho-Rpb1 CTD (S2) | Cell Signaling | #2629 |
| phospho-Rpb1 CTD (S5) | Cell Signaling | #13523 |
| phospho-Rpb1 CTD (S2/5) | Cell Signaling | #13546 |
| SetD2 | Cell Signaling | #80290 |
| RPA70 | Cell Signaling | #2267 |
| γ-H2AX | Cell Signaling | #9718 |
| Smad2/3 | Genetex | GTX111123 |
| RPA32 | Genetex | GTX70243 |
| CDK9 | Genetex | GTX107758 |
| Mre11 | Genetex | GTX118741 |
| Rad50 | Genetex | GTX61150 |
| Nbs1 | Genetex | GTX111123 |
| CtIP | Genetex | GTX70284 |
| RIF1 | Genetex | GTX85718 |
| Ku70 | Genetex | GTX101820 |
| Ku80 | Genetex | GTX109935 |
| AID | Genetex | GTX127276 |
| Xbp-1 | Genetex | GTX102229 |
| 53BP1 | Novus | NB100-304 |
| H3 | Abcam | ab1791 |
| H3K4me3 | Abcam | Ab12209 |
| H3K36me2 | Abcam | Ab9049 |
| H3K36me3 | Abcam | Ab9050 |
| H4K20me2 | Abcam | Ab9052 |

**S2 Table. List of qPCR primers used in this study.**

| PHRF1 | F 5’- ACCCGACTTTTTGTGAGGTG-3’  R 5’- ACCAGGGACCGTACACTCTG-3’ |
| --- | --- |
| AID | F 5’-TCAGCCTGAGGATTTTCACC-3’  R 5’-GCCGAAGTTGTCTGGTTAGCC-3’ |
| LEF1 | F 5’- TCACTGTCAGGCGACACTTC-3’  R 5’- TGAGGCTTCACGTGCATTAG-3’ |
| SetD2 | F 5’- TGCAGCCGTGACTTCAATAG-3’  R 5’- GATCTTCCCCTCAGGGTCTC-3’ |
| Smyd2 | F 5’- CTACCCCGTGTACTCCCTCA-3’  R 5’- GTGGCTCTCAATCTCCTGCTT-3’ |
| Smyd5 | F 5’-GGACCGGGAACAACTAGACA-3’  R 5’- ATTTCCTCGCCTGGCTTAAT-3’ |
| Spt4 | F 5’- GGAGGAACCTGTGTTGTCGT-3’  R 5’- CGCAATGATTCCATCAAATG-3’ |
| Spt5 | F 5’- GACTGGTTTGCCAAAAGGAA-3’  R 5’- CTCTGACAGTGTGGGCTTCA-3’ |
| Spt16 | F 5’- CAGGAATGAGGGCAACATCT-3’  R 5’- TGATCACCAGCGAGTCTTG-3’ |
| Trp53inp1 | F 5’- GTGAGGCGAGTTGTGGAAAT-3’  R 5’- CGACGGAGACCATTTCTGTT-3’ |
| Trp73 | 5’- CAAAGTGTCCACACCACCAC-3’  R 5’- CATACGGCACAACCACACTC-3’ |
| NELF-A | F 5’-GGATGTGATCCAGATCAAGC-3’  R 5’-TGGTCATAGGCTTGTACTTC-3’ |
| NELF-D | F 5’-AAGTCCACGTCAAGGCTATCG-3’  R 5’-GGAAACCGAATGCACTGATAAA-3’ |
| PARP1 | F 5’-ACCACCAACTTTGCTGGCAT-3’  R 5’-CATGTTTCCAAGGGCAACCT-3’ |
| GAPDH | F 5’-TGTGTCCGTCGTGGATCTGA-3’  R 5’- CCTGCTTCACCACCTTCTTGA-3’ |
