# Supplementary material for: PHRF1 promotes the class switch recombination of IgA in CH12F3-2A cells
Source: PLoS One. 2023 Aug 4;18(8):e0285159. doi: 10.1371/journal.pone.0285159 (PMC10403053; doi:10.1371/journal.pone.0285159)
Supplement: Attachment — Submitted filename: Response to reviewer comments.docx [file pone.0285159.s002.docx]

**Reviewer #1:**

Major concerns:
**Q1.** The quality or resolution of all of figures.
The resolution of all these figures is too low to get effective and detailed information. I can’t get any useful information from Figure 2A. The titles of vertical axis on Figure 3B were very difficult to distinguish. The percentage of cells labeled on top-left corner on Figure 4A were also very fuzzy. So, please provide high resolution figures on revised version.

**A1.** Thanks for the reminder. We have improved the resolutions of all figures and reorganized Fig 2. The original Fig 2A has been replaced with S4 Fig and fonts for the percentage of cells in Fig 4A has been enlarged.

**Q2.** Although RNA-seq results were still needed to be further investigated, the validation of this raw transcriptomic data is required for publication to ensure these data were solid and repeatable. Typically, the validation method is that more than 10 genes’ expression in RNA-seq should be validated by RT-qPCR.

**A2.** Thanks for the suggestion. We have completed 14 genes’ expression using RT-qPCR and the result was shown in Fig 4B. Most of these genes were significantly down-regulated, which was in line with the RNA-seq results.

**Q3.** Quantification of blots on Figure 3A. The western blot results on Figure 3A are need to repeat and statistical analysis for quantification, especially for PARP1, γ-H2AX, NELF-A, NELF-D, and H3K36me2/me3 (significantly changed proteins).

**A3.** Thanks for the suggestion. Three independent immunoblots were conducted. Quantification of immunoblots were shown in Fig 3B. The protein levels of PARP1, NELF-D, and H3K36me3 were significantly changed, but NELF-D and γ-H2AX were not statistically affected.

**Q4.** The experimental design for Figure 3C was inappropriate. In “Input” part, pS2, pS2S5 and pS5 should be included to indicate these proteins in WT and KO cell extracts were equal. Rpb1’s blot should also be included in IP results as a control.

**A4.** Thanks for the suggestion. We have added the inputs of pS2 and pS5 of Rpb1 in Fig 3C. Rpb1’s blot was also included in IP result.

**Q5.** The innovation of this study. As mentioned by the authors in end of discussion, the molecular explanation of this study is too “obscure”. In current study, the authors performed lots of molecular experiments, but no reasonable mechanism raised. A molecular explanation is required, at least can partially explain these phonemes. A suggestion may enhance this study’s innovation mentioned below:
Base on the results from CH12F3-2A cells and CD19-Cre mice, PARP1, NELF-A and NELF-D seem to play important roles in PHRF1 regulating CSR process. I do not insist on the following hypothesis but as a possible illustration of how the current results might be interpreted: PARP1, NELF-A or/and NELF-D maybe the key down-stream effectors in PHRF1 regulating CSR progression pathway. Obviously, we need more experiments (express or co-express PARP1, NELF-A and NELF-D in PHRF1 KO cells, then test whether it can rescue or partially rescue the CSR defect) to support the hypothesis. The hypothesis I mentioned is just a possibility and it is more than welcome if you have other regulation mechanism which can be supported by your data. A molecular explanation raised and confirmed by your work would be a shining point in your manuscript.
**A5.** Many Thanks for this suggestion. To highlight the importance of PARP1 and NELF-D in PHRF1 KO cells, we have purchased PARP1 and NELF-D plasmids from Sino Biological. (NELF-A was not available.) PARP1 or NELF-D plasmid was electroporated into PHRF1 KO cells and then IgA switching was determined. The result showed that PARP1 significantly increased IgA production, but NELF-D did not (in Fig 3D). This result indicated that PARP1 might be the main target due to PHRF1 deficiency in PHRF1 KO cells.

Minor concerns:
**Q6.** In Figure S1 panel A, there were two bands on PHRF1’s blot. Please indicate which band is PHRF1 and which is unspecific band.

A6. Figure S1 is moved to Figure 1D; therefore, we have labeled “nonspecific band” in Fig 1D.

**Q7.** The complementary experiment in Figure S1 was a very solid evidence to confirm that PHRF1 KO directly compromised CSR progression. It is highly recommended to move these results to Figure 1.

A7. Many Thanks for this suggestion. We have moved Figure S1 to Figure 1D

**Q8.** The abbreviation of “CIT” should be described in the legend of Figure 1B rather than Figure 1C.

A8. We have added “CIT” in the figure legend of Figure 1B.

Q9. Lack of Data Availability statement.

A9. Data Availability statement is included in the cover letter.

Reviewer #2: 
**Q1.** The logic behind investigating transcriptional elongation in PHRF1 KO cells is not clearly defined. Even though, the PHRF1-deficient CH12 cells showed decreased phosphorylation of Rbp1 subunit of RNA polII at S2 position, the germline transcription at IgH locus in CH12 cells was unaffected. In addition, the effects of PHRF1 deficiency on NELF expression and Rbp1 phosphorylation were totally missing in activated primary B cells. The authors should also examine IgH germline transcription in PHRF1 KO primary B cells.

**A1.** Thanks for the advice. At first we were also confused with this conflict between the decreased pS2 of Rpb1 and unchanged GLTs. Our explanation is that the effect of PHRF1 KO on Rpb1 phosphorylation might “delay” the progression of transcription elongation, but not entirely abolish the elongation process. Therefore, GLTs were not changed in control and PHRF1 KO cells. Germline transcripts of Iμ and Iα in PHRF1 KO primary B cells were shown in S8 Fig. There is no significant difference between control and PHRF1 KO B cells.

**Q2.** Consistent with the role PHRF1 in regulating TGFB signaling, there appears to be a slight increase in the levels of phospho-SMAD2/3 in PHRF1 KO CH12 cells, though this is not quantified. Can the authors test the possibility that the PHRF1-deficent CH12 and primary B cells are differentially sensitive to TGFB stimulation? One can do this by stimulating B cells in varying concentrations of TGFB and measuring the survival, proliferation and CSR to IgA in CH12 and primary B cells.

**A2.** Thanks for this suggestion. We have used different concentrations of TGF-β to treat control and PHRF1 KO primary B cells to address whether TGF-β was a key factor for IgA switching. In S7 Fig, there is no significant change between control and PHRF1 KO B cells in the proliferation using CFSE staining and CSR to IgA under the condition of different concentrations of TGF-β.

Q3. The authors should also provide a more thorough analysis of the transcriptomics data, especially with regards to the transcriptional factors that were identified as being differentially expressed. Perhaps a more careful examination of TGFB signaling could provide readers some more context.

**A3.** We have completed 14 genes’ expression using RT-qPCR and their results were shown in Fig 4B. Most of these genes were significantly down-regulated, which was in line with the RNA-seq results. To further address whether PHRF1 deficiency affected TGF-β signaling, we clustered two heatmaps containing entire components or fold change >2 in TGF-β signaling (KEGG#04350) in S4 Fig. The P-value of TGF-β signaling was 0.56 in the pathway analysis, suggesting that PHRF1 KO might not affect TGF-β signaling.

Q4. The authors observed a slight but non-significant change in the microhomology usage. At what time point were these experiments performed. The repair of switch junctions through MMEJ follows much slower kinetics (PMID: 31806351) and therefore it is better to analyze the microhomology usage at 72 hours post-CIT stimulation. More clones should be screened to calculate the statistical significance of these findings.

**A4.** We isolated DNA from control and PHRF1 KO cells at 72 h post CIT treatment. We collected more clones (control n = 41; PHRF1 KO n = 37) to analyze MH. The results shown in Fig 2 and S4 Fig suggested that PHRF1 KO did not affect the length of microhomology.

Q5. The authors should refine the writing at several places in the manuscript.

**A5.** Thanks for the reminder. We invited Ms. Adaobi Amanna to improve our writing in this revised manuscript.

Q6. The statistical methods used in the study are not detailed enough. The authors should clearly describe the statistical methods used with each figure legend.

**A6.** We used the student’s t test and add it to each figure legend.
